# Carpe diem or carpe mañana? Emotion priming affects intertemporal choice among Internet addicts and normal Internet users

**DOI:** 10.3389/fpsyg.2022.994778

**Published:** 2022-11-17

**Authors:** Hongxia Li, Wen Shan

**Affiliations:** ^1^School of Labor Economics, Capital University of Economics and Business, Beijing, China; ^2^S R Nathan School of Human Development, Singapore University of Social Sciences, Singapore, Singapore

**Keywords:** Internet addiction, Hope Theory, emotion priming, subjective value, intertemporal choice

## Abstract

In this digitalized era, Internet addiction has been a severe problem that needs imperative solutions derived from the same mechanism that leads to its addiction. To uncover a more nuanced mechanism for Internet addiction in association with decision-making focus and emotions and thus generate effective interventions, we conducted three experiments to investigate how various forms of emotion priming affect intertemporal choice among Internet addicts and normal Internet users. We divided the emotions into three categories, namely emotional valence (negative and positive emotions), expected emotion type (expected regret, expected joy), and current emotion type (current regret, current joy). In experiment one, we examined the effect of two participant types (Internet addicts and normal Internet users) with three emotion valences (positive, negative, and neutral). In experiment two, we examined the effect of two participant types (Internet addicts and normal Internet users) with three current emotion types (current regret, joy, and neutral). In experiment three, we examined the effect of two participant types (Internet addicts and normal Internet users) with two expected emotion types (expected regret and expected joy). We conducted a completely randomized experimental design in each experiment and used subjective value as the dependent variable index of intertemporal choice. The results showed that the subjective value of Internet addicts was significantly lower than that of normal Internet users across three studies. The subjective value of individuals primed with positive emotions was significantly higher than those primed with negative emotions, no matter whether they were normal Internet users or addicts (experiment one). The subjective value of individuals primed with expected joy was significantly higher than those primed with expected regret, no matter whether they were normal Internet users or addicts (experiment three). When primed with current joy, however, the Internet addicts' subjective value was significantly lower than when primed with current regret, but this did not apply to normal Internet users (experiment two). These results suggest positive emotions and expected joy enhanced long-term goals and greater rewards focus on intertemporal decision-making compared to negative emotions and expected regret. However, current joy facilitated short-term goals, and smaller rewards focus on intertemporal decision-making compared to current regret. The theoretical and practical implications for Internet addiction are also discussed in this paper.

## Introduction

In this era of the digital revolution, more and more people are addicted to the Internet, greatly affecting their work and life (Amichai-Hamburger and Hayat, 2011; Li et al., 2016). As of April 2022, there were more than five billion internet users worldwide, which is 63.1 percent of the global population (Statista, 2022). Among the massive scale of Internet users, there is a large group of Internet addicts who make decisions focusing on short-term interests, such as escaping reality, at the expense of long-term interests, such as study and work (Schiebener and Brand, 2017; Li et al., 2019; Li, 2021a,b). There is no uniform standard for the definition of Internet addiction. Wei et al. (2010) explored the concepts of Internet addiction and found that Internet addicts were characterized by compulsive Internet use and excessive Internet use (Wei et al., 2010). By far, the most widely used concept of Internet addiction is that of Young. After comparing the concepts and diagnostic criteria of drug addiction, she defined Internet addiction as a kind of impulse control disorder under a non-drug effect (Young, 1998b). In this study, we follow Young's definition of Internet addiction and diagnostic criteria, which assumes that the concept of Internet addiction reflects the three essential characteristics of addiction, namely impulsivity, loss of self-control, and persistence in doing things even though they know there are negative consequences (Weinstein et al., 2014).

Although it is widely known that Internet addiction affects normal work and life in terms of harming physical health, academic performance, and interpersonal relationships (Tsai et al., 2009; Weinstein et al., 2014), Internet addiction is very difficult to quit (Shaw and Black, 2008). Therefore, it is imperative for scholars to investigate the mechanism behind Internet addiction and suggest relevant solutions based on the mechanism. Scholars have various perspectives on explaining Internet addiction. For example, the emotion-related perspective states that the motivational driver for Internet addiction is to suppress negative emotions while enhancing positive emotions (Young, 1998a), and ironically, internet addiction is found to concurrently increase the negative emotional state and decrease the positive emotional state (Longstreet et al., 2019). On the other hand, the decision-making-related perspective specifically assumes that Internet addiction is due to individuals' choice of immediate reward and preference for short-term rewarding behaviors despite negative long-term consequences (Bechara, 2005; Goldstein and Volkow, 2011). Integrating these two perspectives, the current research is the first to uncover a more nuanced mechanism of Internet addiction by systematically investigating the influence of emotions on the intertemporal choices of Internet addicts. Specifically, we investigated how and why various forms of emotions affect intertemporal decision-making in terms of the focus on short-term vs. long-term interest among Internet addicts and normal Internet users. Instead of focusing on the chronic traits of Internet addicts (Ko et al., 2010), in the current study, we focus on contextual situations in terms of primed emotions. This could further shed light on the potential practical interventions for Internet addiction.

## Literature review

Intertemporal decision-making refers to the process in which people weigh and choose the value of events or results occurring at different time points (Prelec and Loewenstein, 1991; Frederick et al., 2002; Duan et al., 2017). To explain the phenomenon of intertemporal choices, many theories have been proposed, including the Construction Level Theory (Liberman and Trope, 1998, 2003; Liberman et al., 2002; Li et al., 2016), the Double-Processing Model (McClure et al., 2004, 2007; Li et al., 2019), the Hot and Cold System Model (Loewenstein, 1996; Metcalfe and Mischel, 1999), and the Self-Control Theory (Laibson, 1997; Fudenberg and Levine, 2006). All these theories explain how individuals' emotional and cognitive systems interact to influence the outcomes of intertemporal choice. However, there has been little research systematically examining the effect of emotion on intertemporal choices.

Previous research has shown that emotions can greatly impact individual attitudes and behaviors, especially decision-making (Lerner and Keltner, 2001; Li et al., 2011; Wang et al., 2021; Karen et al., 2022). A considerable amount of studies have shown that emotions can influence people's decision-making behaviors (Loewenstein and Lerner, 2003; Bechara and Damasio, 2005; Clore and Huntsinger, 2007; Sohn et al., 2015; Herman et al., 2018). For example, Pyone and Isen (2011) induced positive emotions in participants through pictures and words and found that participants with positive emotions chose delayed options with greater rewards in intertemporal decision-making as compared with participants with neutral emotions. Jiang et al. (2022) induced both positive and negative emotions and found that the participants in the positive condition preferred the delayed reward more than the participants in the negative and neutral conditions.

However, these studies only examined the influence of positive and negative emotions on decision-making without considering the other more nuanced patterns of emotion-related categories or examining the influence of emotions related to decision-making itself on intertemporal choice. Integrating the existing literature, we contend that emotions that influence decision-making can be categorized into three categories. The first is the effect of emotional valence (i.e., negative emotion and positive emotion) on decision-making (Johnson and Tversky, 1983). The second category is the influence of the current emotions (e.g., regret and joy) associated with the decision itself (Daniela et al., 2010). The third category is the impact of expected emotions (e.g., expected regret and expected joy) on decision-making (Simonson, 1992). To further uncover the mechanism of Internet addictions, we systematically investigated the influence of these three types of emotions on intertemporal decision-making.

These three categories of emotions are closely related to intertemporal choice. Firstly, from the perspective of emotional valence, the differences in emotional valence before decision-making have an impact on intertemporal choices (Pyone and Isen, 2011; Liu et al., 2013). Specifically, positive emotions can reduce the delay discounting rate of individuals and make them prefer long-term options, whereas negative emotions can increase individuals' delay discounting rate and make them prefer short-term options (Ifcher and Zarghamee, 2011). Secondly, previous studies showed that the emotions associated with decision-making could influence the outcomes of people's intertemporal choices (Daniela et al., 2010). In general, joy and regret are the two emotions that people focus on in the research relating to decision-making. When people feel regretful, they don't like to wait, preferring smaller rewards that are immediately available. However, when they feel joy, they are more likely to wait longer for a bigger reward (Daniela et al., 2010). Lastly, Loewenstein and Lerner (2003) pointed out that many people's decision-making behaviors often involve the prediction of future emotional experiences. The emotions generated by the anticipated outcome of a decision also influence people's intertemporal choices (Hardisty and Weber, 2009; Peters and Büchel, 2010). One study shows that the emotional valence of imagining future events affects the outcome of intertemporal choice (Liu et al., 2013). Specifically, the participants who imagined a future positive emotional event showed a willingness to wait longer for a larger reward than the control group who did not imagine the event. Participants who imagined a future negative emotional event were more likely to choose the immediate but smaller reward than the control group who did not imagine anything. However, there was no significant difference between those who were asked to imagine a neutral emotional event in the future and the control group who did not imagine anything (Liu et al., 2013).

To explain these influences of emotion on intertemporal choices, scholars rely on several theories, including Affect-As-Information Theory (Schwarz and Clore, 1983; Clore et al., 2001), Motivational Dimensional Model of affect (Gable and Harmon-Jones, 2010), and Appraisal-Tendency Framework (Lerner and Keltner, 2001). Affect-As-Information Theory emphasizes the influence of information contained in the different valence of emotions on intertemporal decision-making. Motivational Dimensional Model emphasizes the important role of the emotional motivation dimension in intertemporal decision-making. Appraisal-Tendency Framework emphasizes the influence of emotion-related evaluation dimensions on intertemporal decision-making (Jiang et al., 2022). However, these theories only treat emotion as an informational signal in the process of decision-making but do not consider the mechanism of how emotions interact with individual differences and its consequential influence on individuals' intertemporal decision-making. Hence, in the current study, we introduce the Hope Theory to explain why emotions, especially positive emotions, serve as a guide to lead individuals to make better decisions in the long run.

Hope Theory states that the level of hope can affect an individual's physical and mental health, problem-solving abilities, stress, etc. (Zhang and Zheng, 2002). Hope is defined as a positive motivational state that is based on the interaction between the drive to succeed (i.e., the energy directed toward the goal) and the path (i.e., the plan to achieve the goal) (Snyder et al., 1991b). Path refers to the methods and strategies to achieve goals, while motivation refers to the level of motivation to motivate and maintain individuals to use methods and strategies to achieve goals (Snyder et al., 1991b). The Hope Model mainly contains three components: goals, pathway thinking, and agency thinking (Snyder et al., 2002). First, the value of the goal determines the individual's efforts to achieve the goal. An individual with a high level of hope must have a clear goal because he or she has a clear path and a strong motivation to achieve it (Snyder et al., 2002). Second, once the goal is generated, the method and plan to achieve the goal will also spontaneously form in the mind, which is path thinking. Individuals with high levels of hope have a more specific attitude and come up with more paths to achieve their goals than individuals with low levels of hope (Snyder et al., 1991b, 1996; Irving et al., 1998). In the end, once the goal is set, path thinking has mapped out the way to achieve the goal, and how to use those ways to achieve the goal depends on dynamic thinking. Dynamic thinking is the motivational component of hope and refers to the spirit of striving along a path to achieve a goal, also known as willpower (Magaletta and Oliver, 1999). As such, according to Hope Theory, emotion is the product of hope. For individuals with high hope, their perception of the path and motivation to achieve the goal are clear and strong, so their emotional state is positive. While for individuals with low hope, their perception of achieving the goal is vague and hesitant, so their emotional state is negative (Snyder et al., 1991a).

According to the Hope Theory, therefore, it is speculated that individuals in a positive emotional state will determine their goals earlier. Once they determine their goals, they will plan the path to achieve the goals and come up with more paths. They are also motivated to follow the path of their goals and can overcome obstacles to reach their goals. So, they are more likely to resist the temptations of the present and choose the bigger rewards for which they will have to wait for some time. On the contrary, for those in a negative emotional state, the goal is set later, and the path to achieving the goal is vague. They are more likely to give up the goal and stop. As a result, they often give in to immediate gratification at the expense of the implementation of long-term goals and the greater rewards that long-term goals can bring. At the same time, previous research has found that individuals who are addicted are more likely to make impulsive decisions on intertemporal choice tasks, such as heroin addiction (Harty et al., 2011), drug abuse (Bickel and Marsch, 2002), gambling addiction (Madden et al., 2011), overeating (Stein et al., 2017), and Internet addiction (Li et al., 2019). These addicts tend to have higher delay discounting rates and lower subjective scores for intertemporal choice.

Integrating the Hope Theory and the Internet Addiction literature, in this study, we contend that positive emotional states (operationalized as positive emotions, current joy, and expected joy) are closely associated with individuals' high levels of hope. On the contrary, negative emotional states (operationalized as negative emotions, current regret, and expected regret) are closely related to individuals' low levels of hope. Therefore, we hypothesized that these positive emotional states will enable individuals to make long-term rational decisions. Otherwise, those negative emotional states can make individuals unwilling to wait and make a short-sighted decision regardless of long-term benefits. We propose that such effects will apply to both normal Internet users and Internet addicts.

To test this overall hypothesis and generate potential interventions for Internet addiction, we did not focus on individual traits in emotions but instead adopted the priming method to examine the dynamic effect of emotional states on intertemporal choices among Internet addicts and normal Internet users. The priming method is a commonly used experimental method in social psychology (Hong et al., 2000; Norasakkunkit and Kalick, 2009) and in psychological research, it is mainly the picture priming method (Hong et al., 2000) and the text material priming method (Brewer and Gardner, 1996). Under the condition of the picture priming experiments, the participants are presented with a series of pictures associated with the priming content. Then the impacts of these pictures' content on participants' psychological states and behaviors are tested. In text materials priming, the participants are presented with a text situation, and the participants are asked to write a paragraph of content according to the situation and requirements, which could have an impact on the results of the subsequent experimental task. Although some researchers believe that priming methods increase the rate of false positives (Simmons et al., 2011), others argue that the effectiveness of the priming method can be increased by using some principles in the priming method process (Papies, 2016; Requero et al., 2021; Ueberholz and Fiocco, 2022). These principles are as follows: (1) targeting individuals who value priming goals, (2) activating their specific motivation, (3) providing effective cues, and (4) drawing attention at the appropriate time. Finally, (5) An effective goal-directed behavior needs to be understood and approached by the initiating individual (Papies, 2016). Therefore, we used these two priming methods in our experimental manipulations and strictly followed these five principles in the current study. We used the instruction for the task to target individuals who value priming goals, aroused the participant's motivation by putting the subject in a certain emotional state, used effective emotional pictures and context, drew attention for enough time period, and elicited certain emotions from the individual in accordance with the guiding goals.

The most typical research paradigm of intertemporal choice is the delay discounting paradigm, and the current subjective value and delay discounting rate are widely-used indicators in intertemporal choices (Li et al., 2016; O'Hora et al., 2016). A low subjective value indicates that individuals are short-sighted, and thus it is easier for them to choose the smaller reward available to them when making decisions. On the contrary, a higher subjective value indicates that individuals are less impulsive, and thus when choosing, they are more willing to choose to get a bigger reward after waiting for a certain period (Kirby et al., 1999). In the meanwhile, we used the delay discounting paradigm to study intertemporal choices and used the subjective value of intertemporal decision as our dependent variable indicator. According to the classification of emotions mentioned in the current study, we conducted three experiments successively to explore the effect of emotional priming on the intertemporal choice among Internet addicts and normal Internet users.

## Experiment one

In this experiment, we aimed to investigate the influence of different emotional valences on the subjective value of intertemporal decisions by priming emotional valence. Two sub-hypotheses were devised:

1) The subjective values of intertemporal choice among Internet addicts will be significantly lower than those of normal internet users.
2) The subjective values of intertemporal choices after being primed with positive emotion will be significantly higher than that after being primed with negative emotion.

### Experimental design

The present experiment employed a two-participant type (Internet addicts and normal Internet users) with three valences of emotion (positive, negative, and neutral) in a completely randomized experimental design. The dependent variable assessed in this experiment was subjective value in intertemporal decision-making.

### Participants

Fifty-one individuals (10 female, 41 males; mean age = 21.78 years, standard deviation = 1.05) were recruited from an Internet addiction withdrawal school^1^ in Beijing and North China University of Technology, and 51 normal Internet users (31 female, 20 males; mean age = 20.05 years, standard deviation = 0.85), from North China University of Technology. All participants volunteered to participate in the experiment and had not participated in a similar experiment before. The experiment was conducted separately, and a highlighter pen was given to each subject at the end of the experiment.

### Materials

#### The Internet addiction test

Although the participants from the Internet addiction school were diagnosed as Internet addicts, to be more cautious, we did not assume that all participants from the Internet addiction withdrawal school were Internet addicts or that all participants from the North China University of Technology were normal Internet users. To further identify if they are Internet addicts or not, we used the Internet addiction test. This test is a reliable and valid measure of addictive use of the Internet (Young et al., 1999). The scale consists of 20 items and four factors: “tolerance and time management issues,” “withdrawal reaction,” “interpersonal, health, and academic problems,” and “compulsion and salience.” Respondents rate the applicability of items to themselves using a 5-point Likert-type scale from 1 (*strongly disagree*) to 5 (*strongly agree*). Each item also contained a “not applicable” option. Obtaining a score of 49 or less on the scale indicated that an individual is an average online user who might surf the Web a bit too long at times but who still has control over his or her usage. A score between 50 and 79, on the other hand, meant that the individual experiences occasional or frequent problems due to Internet use. Finally, a score between 80 and 100 denotes a serious addiction to the Internet. Although this scale was published decades ago, more recent research reported a Cronbach's alpha of 0.907 and a split-half reliability coefficient of 0.826, demonstrating that the Chinese version of the scale possessed good reliability and validity (Cao et al., 2010).

#### Delay discounting task

The delay discounting task used in this experiment was adopted from Chen and He's research (Chen and He, 2011). The original version was derived from Rachlin and Jones (2008) research. The delay discounting task contains 19 binary-choice items (with A representing current options and B representing “6 months after” as future options). The total monetary reward for the future options was fixed at 1,000 RMB, while the current options ranged from 50–950 RMB, with an increase of 50 RMB between each item. Specifically, the instructions were,

“Imagine a scenario in which you have a choice for either receiving less money right now or a larger amount 6 months later. Which would you choose?

1. (A) Get 50 RMB now; (B) Get RMB 1,000 after 6 months.

2. (A) Get 100 RMB now; (B) Get RMB 1,000 after 6 months.

……

19. (A) Get 950 RMB now; (B) Get RMB 1,000 after 6 months.

The current subjective value (V) of a participant equals the average of the monetary reward of Choice A selected for the first time and the monetary reward of Choice A for the last item. For example, if a participant selects Choice A from the 10th item, then the subjective value of the 1,000 RMB after 6 months equals the average of the 10th item's Choice A (500 RMB) and the 9th item's Choice A (450 MRB), therefore, 475 RMB. If a participant selects Choice A for all items, then the subjective value is 25. If a participant selects Choice B for all items, then the subjective value is 975. Thus, the higher the subjective value is, the stronger the long-term focus with greater rewards (the less impulsivity) is during decision-making.

#### Pictures of emotion valence priming

We selected 20 positive emotion pictures (Valence: 6.82 ± 0.70; Arousal: 6.03 ± 0.83), 20 negative emotion pictures (Valence: 2.69 ± 0.66; Arousal: 6.71 ± 0.43), and 20 neutral emotion pictures (Valence: 5.29 ± 0.62; Arousal: 3.45 ± 0.95) from The Chinese Affective Picture System (CAPS) (Bai et al., 2005). The emotion pictures show faces or situations that are associated with certain emotions, whereas the neutral pictures show geometrical graphs. Sample positive emotions are smiling faces; sample negative emotions are scenes of disaster; sample neutral emotions are the triangle. We matched images of the same valence for arousal. The pictures in the different emotion valence priming conditions can well distinguish the positive, negative, and neutral emotions, and the arousal of positive and negative emotions was highly consistent. Moreover, the arousal of positive and negative emotions was higher than the neutral group.

### Procedures

Before participating in the formal experiment, participants completed the Internet addiction test. Based on the results of the scale, we categorized participants into two groups: participants with Internet addiction who scored higher than 49 (hereinafter called Internet addicts) and participants without Internet addiction who scored 49 or below (hereinafter called normal Internet users). The results of the test demonstrated that all but one participant from the Internet addiction withdrawal school scored higher than 49, and thus this one data was deleted^2^. All participants from the North China University of Technology were normal Internet users who scored 49 or below. Upon arrival at the lab, both groups were randomly assigned to one of the emotion valence priming conditions: positive emotion priming (*n* = 39), negative emotion priming (*n* = 34), and neutral condition (*n* = 28). Participants were placed in a quiet room to complete the experiment.

The experiment consisted of two sessions. In the first session, all emotion pictures were randomly presented by using the E-prime 2.0 software on a normalized computer screen. The participants clicked “start” once they understood the experimental tasks and requirements. A “+” of fixation lasting for a half-second was presented in the center of the screen. Then, a blank appeared, lasting for 500 ms. After the blank disappeared, the emotion pictures appeared on the screen lasting for 4 sec. In this way, all 20 emotion pictures were shown one by one in a randomized order. After the last emotion picture disappeared, it was followed by two questions on a scale from 1 (*slight or without*) to 5 (*very strong*): (1) to what extent the pictures described positive emotions, indicating positive emotional intensity, (2) to what extent the pictures described negative emotions, indicating negative emotional intensity. This evaluation served as the manipulation check. During the experiment, the participating time was not limited, and a blank again appeared, lasting for 500 ms after the participant responded. Then, the next session started.

In the second session, the participants were asked to finish the delay discounting task on the computer. After finishing the delay discounting task, all participants were told to complete their demographic information.

### Results

#### Manipulation checks

Experimental data obtained in this study were analyzed by using SPSS 16.0 software. We conducted a two-participant type (Internet addicts and normal Internet users) with three valences of emotion (positive, negative, and neutral) ANOVA, with the intensity of positive and negative emotions as the dependent variables. The results showed that the priming was effective. Specifically, the main effect of emotion valence priming on the positive emotional intensity was significant, *F*_(2, 95)_ = 2861.674, *p* = 0.000, η^2^ = 0.948. The positive emotional intensity was significantly higher when primed with positive emotion (*M* = 4.922, *SD* = 0.035) than the neutral condition (*M* = 3.000, *SD* = 0.041), which in turn, was significantly higher than when primed with the negative emotion (*M* = 1.059, *SD* = 0.037). Similarly, the main effect of emotion valence priming on the negative emotional intensity was also significant, *F*_(2, 95)_ = 1,795.545, *p* = 0.000, η^2^ = 0.974. The negative emotional intensity was significantly higher when primed with the negative emotion (*M* = 4.971, *SD* = 0.047) than the neutral condition (*M* = 2.895, *SD* = 0.052), which in turn, was significantly lower than when primed with the positive emotion (*M* = 1.103, *SD* = 0.044).

In addition, the Internet addicts and normal Internet users did not significantly differ in positive emotional intensity, *p* = 0.948, or negative emotional intensity, *p* = 0.995. The interactions between participant type and emotion valence priming on both positive and negative emotional intensity were also not significant, with *p* > 0.05.

#### Dependent variables analysis

An ANOVA of two participant types (Internet addicts and normal Internet users) with three emotion valence priming (positive, negative, and neutral) was conducted with subjective value as the dependent variable. The descriptive statistics of Internet addicts and normal Internet users' subjective values in different emotion valence priming conditions are shown in Table 1.

**Table 1:** Internet addicts and normal internet users' subjective values in different emotion valences *(M* ± *SD)*.

**Participant type**	**Emotion valence**	**Subjective value**	**N**
Normal Internet users	Positive	784.21 ± 180.32	19
	Negative	655.88 ± 217.86	17
	Neutral	796.67 ± 208.28	15
Internet addicts	Positive	737.50 ± 231.06	20
	Negative	501.79 ± 189.20	17
	Neutral	734.62 ± 144.89	13

**Figure 1:**
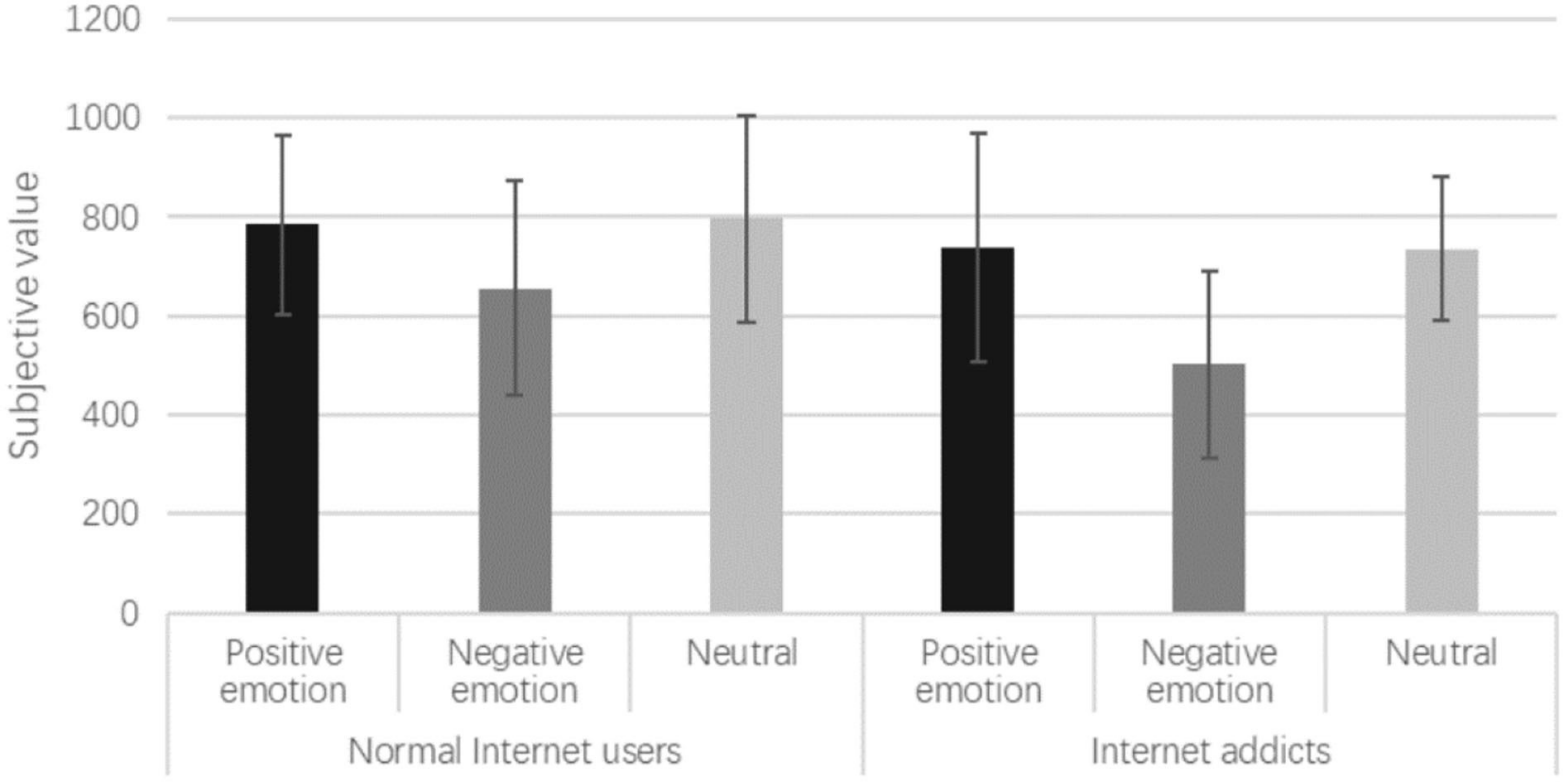
The main effects of participant type and emotion type.

Supporting sub-hypothesis one, the results showed that the subjective value of Internet addicts was significantly lower than that of normal Internet users [*F*_(1, 95)_ =5.688, *p* = 0.019, η^2^ = 0.056]. The main effect of emotion valence priming on the subjective values was significant [*F*_(2, 95)_ = 10.586, *p* = 0.000, η^2^ = 0.182]. Specifically, for both Internet addicts and normal Internet users, the subjective values following the positive emotion priming were significantly higher than those following the negative emotion priming (*p* < 0.001). This result supports sub-hypothesis two. In addition, the results also showed that the subjective values following the negative emotion priming were significantly lower than those in the neutral control condition. However, there was no significant difference between positive emotion priming and neutral control conditions (*p* = 1.000). No significant interaction was found between the participant type and emotion valence priming (*p* = 0.284). The results are shown in Figure 1.

### Discussion

The results first demonstrated that the subjective values of the intertemporal choice of Internet addicts were significantly lower than normal Internet users. This result is consistent with existing literature focusing on different types of addict groups (Bickel and Marsch, 2002; Harty et al., 2011; Madden et al., 2011; Li et al., 2016; Stein et al., 2017). Second, regardless of whether Internet addicts or not, individuals primed with positive emotion had higher subjective values of the intertemporal choice than those primed with negative emotion. This is consistent with previous studies on the influence of emotions on intertemporal choices (Ifcher and Zarghamee, 2011; Pyone and Isen, 2011; Liu et al., 2013). In other words, positive emotions made individuals more willing to wait for a period to get a bigger reward, as compared to negative emotions. In addition, the subjective values of individuals primed with negative emotions were significantly lower than those primed with positive and neutral emotions. This indicates that negative emotions led individuals to be more willing to get a smaller reward immediately and to make short-sighted choices, even when compared with neutral emotions. These results support our overall hypothesis that positive emotional states (operationalized as positive emotions in this experiment) enable individuals to make long-term, rational decisions, whereas negative emotional states lead individuals to make short-term, impulsive decisions. Using a more nuanced perspective, these results show that the valence of emotion significantly impacts people's intertemporal choices (Clore, 1992; Loewenstein and Lerner, 2003; Bechara and Damasio, 2005; Clore and Huntsinger, 2007).

However, no significant difference between the subjective values was found between the positive emotion priming condition and the neutral priming condition, which is inconsistent with Ifcher and Zarghamee (2011) study. One of the reasons may be the difference between the experimental materials, namely movie clips used in the previous study (Ifcher and Zarghamee, 2011) and emotion pictures in the current study.

## Experiment two

According to previous studies, we found that when individuals make decisions, they are often accompanied by two kinds of emotions related to the decision, namely regret and joy (Daniela et al., 2010). When people feel regret, they do not like to wait and thus prefer smaller rewards that are immediately available. However, when people experience joy, they are more likely to wait longer for a bigger reward (Daniela et al., 2010). Therefore, in this experiment, we investigated the influence of the type of emotion (i.e., regret vs. joy) on the subjective value of intertemporal choices by adopting the method of priming. Two sub-hypotheses were devised:

3) The subjective values of intertemporal choice among Internet addicts will be significantly lower than those of normal internet users.
4) The subjective values of intertemporal choices after being primed with current joy will be significantly higher than that after being primed with current regret.

### Experimental design

The current study employed a two-participant type (Internet addicts and normal Internet users) with three current emotion types (regret, joy, and neutral) completely randomized experimental design. The participant type and current emotion type were the independent variables. The dependent variable assessed in this study was subjective value.

### Participants

Forty-six individuals (21 female, 25 males; mean age = 19.02 years, standard deviation = 1.03) were recruited from an Internet addiction withdrawal school in Beijing and the North China University of Technology, and 60 normal Internet users (30 female, 30 males; mean age = 19.90 years, standard deviation = 0.90) from North China University of Technology. All participants volunteered for the experiment and had not participated in a similar experiment before. The experiment was conducted separately, and a highlighter pen was given to each participant at the end of the experiment.

### Materials

*The Internet Addiction Test*. Same as experiment one.

*Delay discounting task*. Same as experiment one.

*A priming situation that induces current feelings of regret and joy*. Based on the previous study (Kahneman and Tversky, 1982), we adopted a well-established approach to prime joy or regret from previous research on decision-making. The participants were given a situation of stock selection, including A and B stocks. The situation of stock description included the trend diagram of the opening, closing, highest, lowest, buying, and asking prices of a stock. For the current regret priming condition, no matter which stock is chosen by the participants, the computer showed the feedback according to the preset program. The information about the stock crash was presented to the participants, which induced the participants to have very regretful emotions in their current state. For the current joy priming condition, no matter which of the two stocks they chose, the computer showed the pre-programmed feedback, which was a rapid appreciation of the stock, inducing a joyful mood. For the neutral condition, no matter which stock A or B was chosen by the participants, the computer showed the pre-programmed feedback, which presented some basic descriptions of the stock. All these situations were presented on the computer screen by using E-prime 2.0 software.

### Procedures

Before participating in the formal experiment, participants completed the Internet addiction test. Based on the results of the scale, we categorized participants into two groups: participants with Internet addiction who scored higher than 49 (hereinafter called Internet addicts) and participants without Internet addiction who scored 49 or below (hereinafter called normal Internet users). The results of the test demonstrated that all but two participants from the Internet addiction withdrawal school scored higher than 49, and thus these two data were deleted. All participants from the North China University of Technology were normal Internet users who scored 49 or below.

Upon arrival at the lab, both groups were randomly assigned to one of the current emotion type priming conditions: current regret priming (*n* = 34), current joy priming (*n* = 36), and neutral condition (*n* = 34). Participants were placed in a quiet room to complete the experiment.

The experiment consisted of two sessions. In the first session, the computer screen presented the participants with one of the three current emotion-priming tasks that were related to decision-making, namely, current joy, current regret, or current neutral. Specifically, the instruction was first presented to the participants. After the participants understood the requirements of the experiment, they would click “start” to start the next tasks. On the computer screen, the description of stock A and stock B was presented. Participants were asked to choose one stock to purchase by pressing the button after analyzing the two stocks. If they choose stock A, then they press the numeric key “1.” If they choose stock B, then they press the numeric key “2.” After making the decision, the participants were immediately given feedback according to the preset program that elicits either current joy, current regret, or current neutral. After completing the emotional priming task, the participants were further asked to answer the same two questions as in Experiment one on a scale ranging from 1 (*slight or without*) to 5 (*very strong*): (1) to what extent do you feel joyful currently, indicating joy intensity, (2) to what extent you feel regretful currently, indicating regret intensity. This evaluation served as the manipulation check.

In the second session, the participants were asked to finish the delay discounting task on the computer. After finishing the intertemporal choice task, all participants were told to complete their demographic information.

### Results

#### Manipulation checks

Experimental data obtained in this study were analyzed by using SPSS 16.0 software. We conducted a two-participant type (Internet addicts and normal Internet users) with three current emotion priming types (regret, joy, and neutral) ANOVA, with the current regret and joy intensity emotions as dependent variables. The results demonstrated that the priming manipulation was effective. Specifically, the main effect of current emotion type priming on the joy intensity was significant [*F*_(1, 98)_ = 73.998, *p* = 0.000, η^2^ = 0.594). The joy intensity was significantly higher when primed with current joy (*M* = 4.795, *SD* = 0.164) than when primed with the current regret (*M* = 1.903, *SD* = 0.184), which in turn, was significantly higher than the neutral condition (*M* = 2.824, *SD* = 0.176). Similarly, the main effect of current emotion type priming on the regret intensity was also significant [*F*_(1, 98)_ = 134.859, *p* = 0.000, η^2^ = 0.728). The regret intensity was significantly higher when primed with the regret emotion (*M* = 4.645, *SD* = 0.124) than when primed with the neutral emotion (*M* = 2.765, *SD* = 0.133), which in turn, was significantly higher than the joy condition (*M* = 1.590, *SD* = 0.124).

In addition, the Internet addicts and normal Internet users did not significantly differ in joy emotional intensity (*p* = 0.648), or regret emotional intensity (*p* = 0.352). The interactions between participant type and emotion valence priming on both positive and negative emotional intensity were also not significant with *p* > 0.05.

#### Dependent variables analysis

We conducted a two-participant type (Internet addicts and normal Internet users) with three current emotion type priming (regret, joy, and neutral) ANOVA, with subjective value as the dependent variable. The descriptive statistics of Internet addicts and normal Internet users' subjective values in different emotion-type priming conditions are shown in Table 2.

**Table 2:** Internet addicts and normal Internet users' subjective values in current emotion type *(M* ± *SD)*.

**Participant type**	**Current emotion type**	**N**	**Subjective value**
Normal Internet users	Regret emotion	21	700.00 ± 254.37
	Joy emotion	18	797.62 ± 174.98
	Neutral	21	726.19 ± 172.93
Internet addicts	Regret emotion	13	746.15 ± 154.73
	Joy emotion	18	491.67 ± 217.78
	Neutral	13	707.69 ± 113.37

The results yielded a significant main effect of participant type [*F*_(1, 98)_ = 5.922, *p* = 0.017, η^2^ = 0.057], such that the subjective values of the Internet addicts were significantly lower than the normal Internet users. Therefore, sub-hypothesis three was supported.

**Figure 2:**
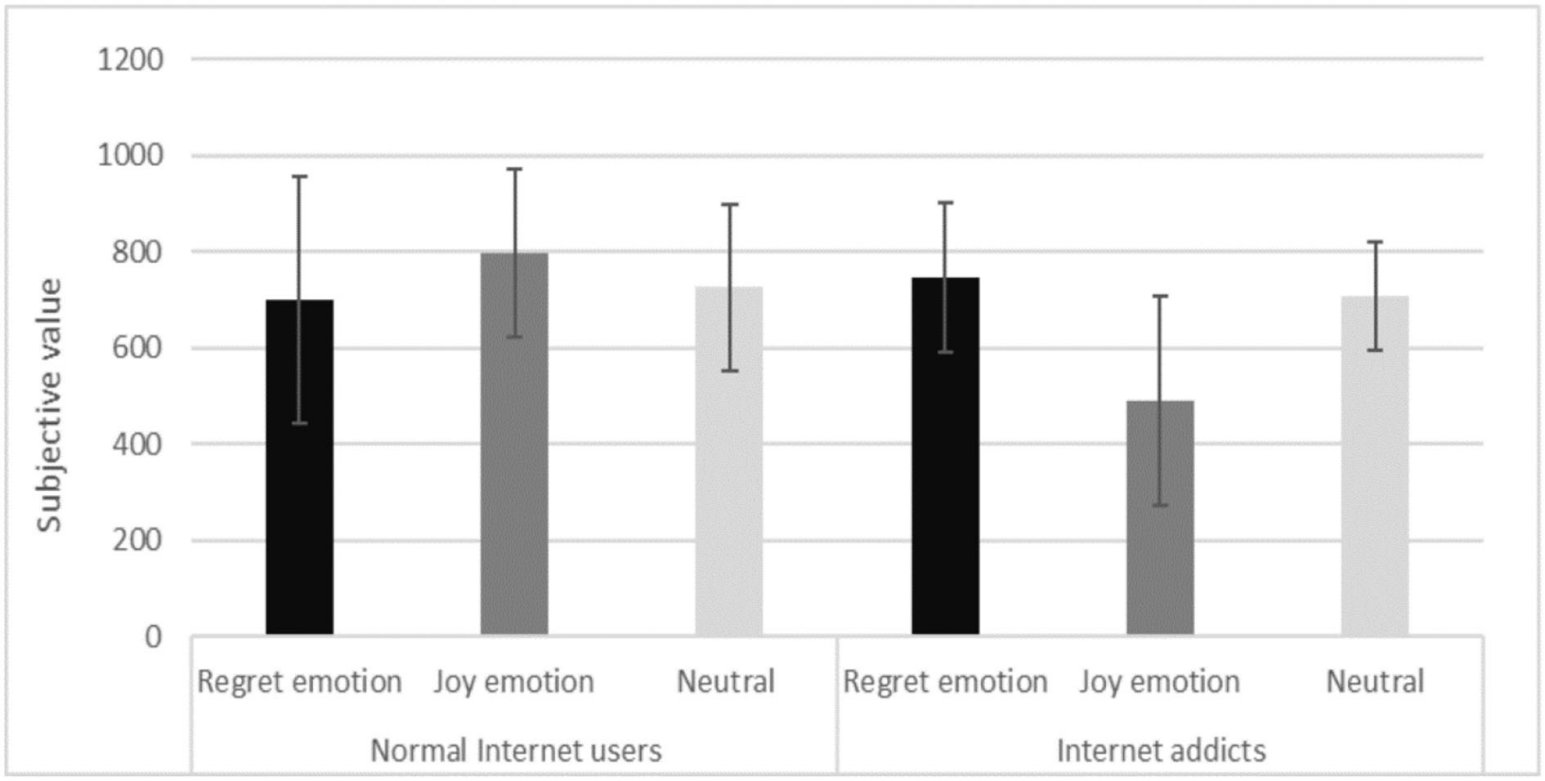
The interaction between participant type and emotion type.

Although no significant effect of current emotion type was found with *p* = 0.159, there was a significant interaction between participant type and current emotion type [*F*_(2, 98)_ = 8.585, *p* = 0.000, η^2^ = 0.149] (as shown in Figure 2). The simple effect analysis indicates that current emotion type priming significantly impacted the subjective values among Internet addicts [*F*_(2, 41)_ = 9.829, *p* = 0.000, η^2^ = 0.324], but this effect was not found among normal Internet users (*p* = 0.292). Surprisingly, among Internet addicts, the subjective values after regret priming were significantly higher than after joy priming. Thus, sub-hypothesis four was contradicted.

### Discussion

The current experimental results first validate sub-hypothesis three (consistent with sub-hypothesis one). As in experiment one, experiment three also shows that the subjective value of the intertemporal choice of Internet addicts was significantly lower than that of normal Internet users. Again, this is consistent with previous addiction research that focuses on other addict groups (Bickel and Marsch, 2002; Harty et al., 2011; Madden et al., 2011; Li et al., 2016; Stein et al., 2017).

It is worth noting that sub-hypothesis four was not supported. Conversely, the results showed that the subjective values among Internet addicts primed with current regret were significantly higher than those primes with current joy. In other words, current joy, surprisingly, facilitated Internet addicts to make more impulse decisions to gain immediate rewards without considering long-term greater rewards. Although this looks counterintuitive, some indirect evidence from previous literature can help explain why. For example, electronic commerce research reveals that a positive mood encourages people to buy, whereas a negative mood suppresses the desire to buy (Rook and Gardner, 1993). More relevantly, people are more willing to shop online and conduct impulse purchasing behavior when they are feeling good at the moment (Madhavaram and Laverie, 2004). Because of this, website design elements can increase the currently perceived enjoyment, which in turn, leads to psychological impulsivity (Parboteeah et al., 2009). This is interesting because although previous research used to associate general negative emotional states with Internet addiction or short-sighted decision-making, current joyful emotion may induce people to think of “carpe diem,” namely to make the most of the present time and give little thought to the future. On the other hand, current regret emotion might let Internet addicts associate with their past regret feelings after they surfed online too much (Young, 2007) and thus elicit a “carpe mañana,” or a “seize-the-future” mindset. These results uncover a more nuanced mechanism of Internet addiction and thus provide a more precise intervention approach.

## Experiment three

The results of experiment one indicated that individuals tended to make more short-sighted intertemporal decisions when primed with negative emotions than when primed with positive emotions. However, experiment two showed that Internet addicts tended to make more short-sighted intertemporal decisions when primed with current regretful feelings, as compared to when primed with current joyful feelings. From these seemingly contradictory results, we cannot tell whether the decisions made by individuals currently with joyful and regretful feelings are simply because of these emotions or whether the participants make decisions based on the cognitive evaluation of certain emotions that are generated by the anticipated outcome of a decision before deciding. In experiment three, to make sense of and reconcile the results of experiments one and two, we aimed to investigate the effects of expected regret and expected joy on intertemporal choice. According to the literature review, three sub-hypotheses were devised:

5) The subjective values of intertemporal choice among Internet addicts will be significantly lower than those of normal internet users.
6) The subjective values following the expected regret priming will be significantly lower than that following the expected joy priming.

### Experimental design

A two-participant group type (Internet addicts and normal Internet users) with two expected emotion types (expected regret and expected joy) completely randomized experimental design was conducted to examine how expected emotions associated with the intertemporal choice might influence the results of intertemporal choice among Internet addicts and normal Internet users. Participant group type and expected emotion priming were the independent variables. The subjective value in the intertemporal choice was the dependent variable.

### Participants

Thirty-three individuals (10 female, 23 males; mean age = 18.48 years, standard deviation = 0.95) were recruited from an Internet addiction withdrawal school in Beijing and North China University of Technology, and 44 normal Internet users (26 female, 18 males; mean age = 19.06 years, standard deviation = 1.01) from North China University of Technology. All participants volunteered for the experiment and had not participated in a similar experiment before. The experiment was conducted separately, and a highlighter pen was given to each participant at the end of the experiment.

### Materials

*The Internet Addiction Test*. Same as experiment one.

*Delay discounting task*. Same as experiment one.

*A priming situation that induces feelings of expected regret and expected joy*. In this experiment, we used the descriptive priming paradigm adapted from previous research (van der Schalk et al., 2012). This task was conducted with paper and pencil, and the priming situations were presented on a sheet of paper. In the expected regret priming condition, the participants were asked to imagine and write down three events in which they expected to feel very regretful after making a decision. In the expected joy priming condition, the participants were asked to imagine and write down three events in which they expected to feel very joyful after making a decision. Both emotion-priming description events were limited to 15 min.

### Procedures

Before participating in the formal experiment, participants completed the Internet addiction test. Based on the results of the scale, we categorized participants into two groups in the same way as in experiments one and two. The results of the test demonstrated that all but two participants from the Internet addiction withdrawal school scored higher than 49, and thus these two data were deleted. All participants from the North China University of Technology were normal Internet users who scored 49 or below.

Upon arrival at the lab, both groups were randomly assigned to one of the expected emotion type priming conditions: expected regret priming (*n* = 37), expected joy priming (*n* = 38), and neutral condition (*n* = 34). Participants completed the experiment in a quiet room.

The experiment consisted of two sessions. In the first session, the experimenter presented the expected emotion priming materials to the participants, namely either expected regret or expected joy. After completing the expected emotion priming task, the participants were asked to answer two questions on a scale from 1 (*slight or without*) to 5 (*very strong*): (1) to what extent do you expect how joyful you will feel after the events you that just described, indicating expected joy intensity, (2) to what extent you expect how regretful you will feel after the events you that just described, indicating expected regret intensity.

In the second session, the participants were asked to complete the delay discounting task on a sheet of paper. After finishing the intertemporal choice task, all participants were told to fill out their demographic information.

### Results

#### Manipulation checks

Experimental data obtained in this experiment were analyzed by using SPSS 16.0 software. We conducted a two-participant type (Internet addicts and normal Internet users) and two expected emotion types (expected regret and expected joy) ANOVA, with the intensity of their expected regret and expected joy emotions as the dependent variables.

The results demonstrated that the priming manipulation was effective. Specifically, the main effect of expected emotion type priming on the expected joy intensity was significant [*F*_(1, 73)_ = 647.586, *p* = 0.000, η^2^ = 0.899]. For both Internet addicts and normal Internet users, the expected joy intensity was significantly higher when primed with expected joy (*M* = 4.757, *SD* = 0.096) than when primed with the expected regret (*M* = 1.316, *SD* = 0.095). Similarly, the main effect of expected emotion type priming on the expected regret intensity was also significant [*F*_(1, 73)_ = 672.759, *p* = 0.000, η^2^ = 0.902]. The expected regret intensity was significantly higher when primed with the expected regret (*M* = 4.474, *SD* = 0.725) than when primed with the expected joy (*M* = 1.108, *SD* = 0.315).

In addition, the Internet addicts and normal Internet users did not significantly differ in expected regret intensity (*p* = 0.251), nor expected joy intensity (*p* = 0.575). The interactions between participant type and expected emotion type priming on both expected regret and joy were also not significant (*p* > 0.05).

#### Dependent variables analysis

We conducted a two-participant type (Internet addicts vs. normal Internet users) with two expected emotion types (expected regret and expected joy) ANOVA, with the subjective value as the dependent variable. The descriptive statistics of Internet addicts and normal Internet users' subjective values in different expected emotion-type priming conditions are shown in Table 3.

**Table 3:** Internet addicts and normal Internet users' subjective values in different expected emotion types (M ± SD).

**Participant type**	**Expected emotion type**	**N**	**Subjective value**
Normal internet users	Expected regret emotion	24	702.50 ± 256.74
	Expected joy emotion	20	854.17 ± 116.95
Internet addicts	Expected regret emotion	13	461.11 ± 198.94
	Expected joy emotion	18	746.15 ± 154.73

The results yielded a significant main effect of participant type [*F*_(1, 71)_ = 15.303, *p* = 0.000, η^2^ = 0.177], such that the subjective values of the Internet addicts were significantly lower than the normal Internet users. Therefore, sub-hypothesis five was supported.

**Figure 3:**
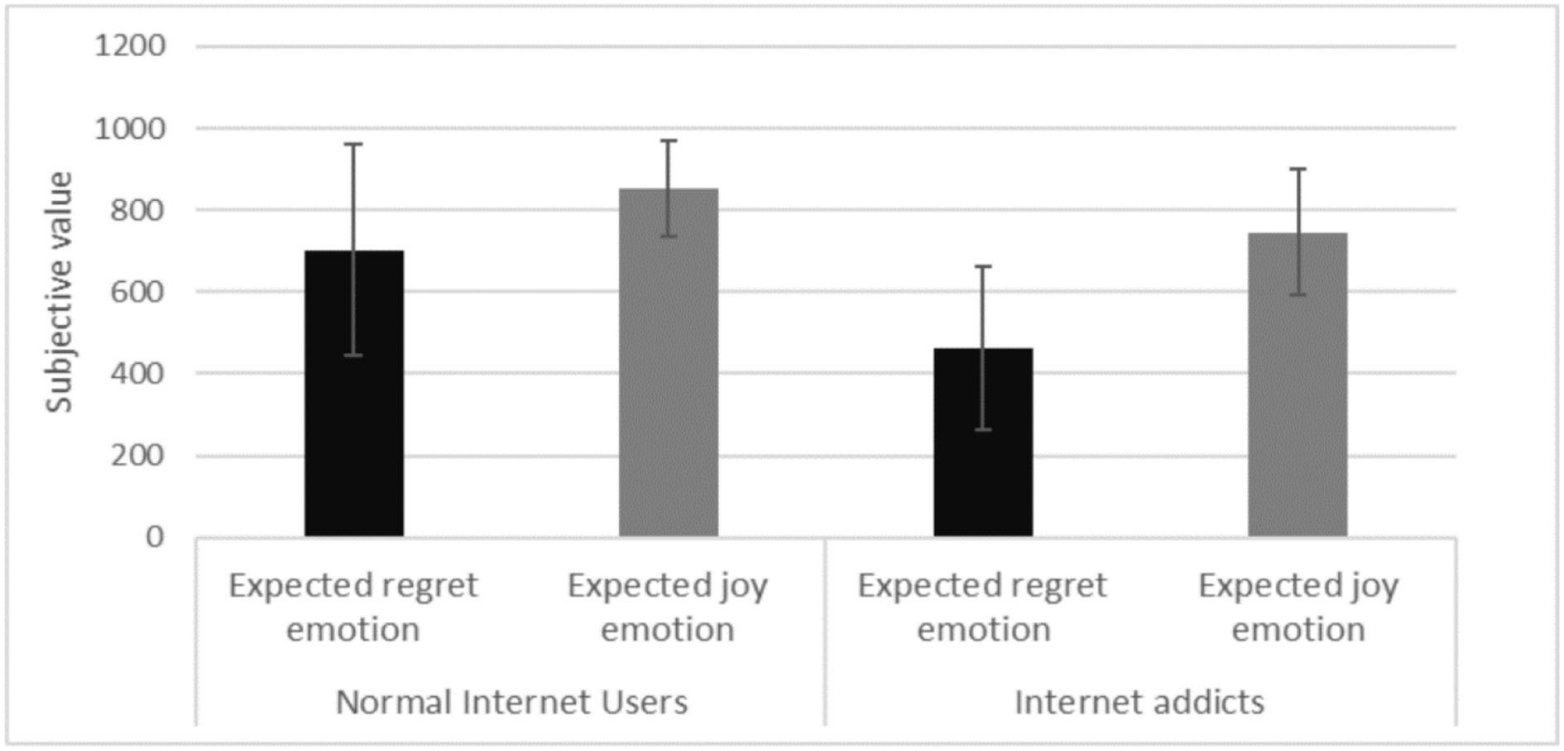
The main effects of participant type and emotion type.

The main effect of expected emotion type priming on the subjective values was also significant [*F*_(1, 71)_ = 23.906, *p* = 0.000, η^2^ = 0.252]. Specifically, for both Internet addicts and normal Internet users, the subjective values following the expected joy priming were significantly higher than those following the expected regret priming. This result supports sub-hypothesis six. No significant interaction between participant type and expected emotion type was found with *p* = 0.140. The results are shown in Figure 3.

### Discussion

The results of Experiment three support both sub-hypotheses. As in experiments one and two, the current experimental results also showed that the subjective values of Internet addicts were significantly lower than that of normal Internet users, which is consistent with the previous studies (Bickel and Marsch, 2002; Harty et al., 2011; Madden et al., 2011; Li et al., 2016; Stein et al., 2017). Furthermore, the subjective value of individuals primed with expected regret was significantly lower than individuals primed with expected joy for both Internet addicts and normal Internet users. This is consistent with previous studies (Daniela et al., 2010). In experiment three, the priming paradigm used could make both normal Internet users and Internet addicts aware of the possible regret or joy after decision-making. After priming the expectation of regret, individuals were more likely to choose the smaller reward that was immediately available.

## General discussion

### Theoretical implications

In the current research, we conducted three experiments to investigate the more nuanced mechanism of Internet addiction by integrating both emotion-related perspectives and decision-making perspectives. Three experiments were conducted to examine how various patterns of emotion priming affected intertemporal choice among Internet addicts and normal Internet users. The results showed that consistent with previous studies (Bickel and Marsch, 2002; Harty et al., 2011; Madden et al., 2011; Li et al., 2016; Stein et al., 2017; Li, 2021a), the subjective value of the Internet addicts was significantly lower than that of the normal Internet users across three experiments.

However, the three experiments showed different results regarding how different categories of emotions affected the participants' intertemporal choices. First, both general positive emotions and expected joy were more likely to encourage individuals to make more long-term, greater-rewarding decisions, as compared to general negative emotions and expected regret. This is consistent with previous studies (Clore, 1992; Lerner and Keltner, 2001; Loewenstein and Lerner, 2003; Bechara and Damasio, 2005; Clore and Huntsinger, 2007; Li et al., 2011). This guidance of positive emotional states on long-term decisions can be explained by the Hope Theory. Based on the emotional view of hope (Snyder et al., 1991a), positive emotions enable individuals to maintain a positive motivational state for success and achieving goals. On the contrary, negative emotions can make individuals maintain a negative motivational state for success and achieving goals. Participants who were primed with general positive emotions or expected joy had a high sense of hope and a higher motivation to achieve a goal, thus enabling them to make long-term decisions with greater rewards in the intertemporal choice. On the other hand, general negative emotions and expected regret imply low hope and a lower motivation to achieve a goal or the expectation that even though they achieve a goal, they will feel regretful. Thus, they can induce individuals to make a more short-sighted decision with smaller rewards in the intertemporal decision tasks.

According to Snyder's hope model (Snyder et al., 2002), individuals with general positive emotions or expected joy give more weight to future goals and motivate themselves to work toward the goals. In the path thinking process, as compared to individuals with general negative emotions or expected regret, individuals with general positive emotions or expected joy are more likely to spontaneously form the method and the plan to achieve the goal in their minds once the goal is generated. In the dynamic thinking process, individuals with general positive emotions or expected joy are more like to achieve the goal once the goal is set. In contrast, individuals with general negative emotions or expected regret are more likely to fail to achieve their goal due to a lack of clarity and motivation on the goal and method and being not good at using dynamic thinking.

Second, it is a surprise that, in experiment two, Internet addicts primed with current joy were more likely to make impulsive, short-term decisions in the intertemporal decision task than those primed with current regret. Although it seems contradictory to the results of experiments one and three, these results of the current emotions' impacts also reveal a boundary effect of the Hope Theory, which focuses on people's cognitive calculation for the future. More importantly, these counterintuitive results shed light on a more nuanced mechanism of Internet addiction. Specifically, it is well-known that Internet addicts are often in a chronic mood of regret after surfing online excessively (Young, 2007). Therefore, when knowing that they chose the wrong stock in a gambling-like task and thus felt regretful, Internet addicts were likely to associate their past regretful feelings due to too much Internet usage. Thus, this regret priming paradigm might make them repent in their hearts and thus avoid making impulsive decisions like before.

On the other hand, when knowing that they chose the right stock in a gambling-like task and thus feeling joyful, this current positive mood might elicit their inertial decision-making. Inertia refers to a decision process that involves repeated usage of a similar effortless information search pattern (Huang and Kuo, 2020). Just like how current positive moods increase impulsive shopping (Rook and Gardner, 1993; Madhavaram and Laverie, 2004), they could also promote inertia in decision-making (Huang and Kuo, 2020). As demonstrated in the three experiments in the current study and previous research (Bickel and Marsch, 2002; Harty et al., 2011; Madden et al., 2011; Li et al., 2016; Stein et al., 2017; Li, 2021a,b), the inertial decision-making for Internet addicts is impulsive short-term decision-making with smaller rewards. This explains why only Internet addicts were likely to make impulsive decisions after being primed with current joy.

### Practical implications

The nuanced mechanism of emotional impacts on intertemporal decision-making can guide the development of potential interventions for Internet addiction. First, general emotional states and expected joy may lead individuals to focus on their future with hope, instead of the immediate gain, in decision-making. This implies that Internet addicts can receive training on developing the mindset of hope for the future so that they can set goals for their studies, careers, or lives, then map out the path or method to reach the goals and motivate themselves to stay on the path with willpower. It will be helpful to visualize their future with joyful images, too. Second, current regret can help Internet addicts stay away from impulsive decision-making, such as surfing online excessively. Therefore, Internet addicts can try to reflect on how their addiction brings negative consequences to their lives and thus elicit regretful feelings, letting them avoid impulsive decisions.

### Limitations and future directions

Although we uncovered the nuanced mechanism of Internet addiction by examining various emotion-priming effects on the intertemporal choice among Internet addicts and normal Internet users, our study has several limitations that can direct future studies. First, we used the Hope Theory to explain the current findings, and these explanations are only based on our theoretical assumptions. The current study did not directly measure hope as a potential mediator, which can be done in future studies. Second, in the current study, we only used the intertemporal choice tasks related to money. However, similar intertemporal choice situations also exist in natural and social environments. Hence, the generalizability of future research will benefit from using various intertemporal choice tasks related to natural and social environments. Third, despite the great reliability, validity, and popularity of the Chinese version of the Internet addiction test, it is still an old scale that might treat Internet addiction as a psychiatric disorder rather than a hypothetical construct. We call for researchers to translate more updated scales to measure Internet addiction in the Chinese language.

## Conclusion

In sum, various patterns of emotion priming affect intertemporal choice differently among Internet addicts and normal Internet users' intertemporal choices. Our findings indicate that the individual's intertemporal choice was influenced by general emotional valence (positive and negative emotion), current emotion type (regret, joy, and neutral), and expected emotion type (expected regret emotion and expected joy emotion). Specifically, normal Internet users made more long-term decisions than Internet addicts. Moreover, individuals in a positive emotional state (i.e., general positive emotion or expected joy) were more likely to make long-term decisions in the intertemporal choice task, whereas individuals in a negative emotional state (general negative emotion or expected regret) were short-sighted in the intertemporal choice task. These results suggest that individuals with a positive emotional state, especially when the emotion is associated with the future, are more likely to increase the values of the goal and strengthen their willpower to achieve their goals through the paths. Nevertheless, Internet addicts who were currently feeling regret were more likely to make long-term decisions than those who were currently feeling joyful. This result suggests that joy can elicit inertial decision-making, which is a short-term focus among Internet addicts. The current study offers a nuanced theoretical basis for understanding intertemporal decision-making and, thus, Internet addiction. This study provides theoretical and practical guidance for individuals to make long-term decisions in various situations through clear goals, specific paths, and strong willpower.

## Data availability statement

The original contributions presented in the study are included in the article/supplementary material, further inquiries can be directed to the corresponding author.

## Ethics statement

Ethical review and approval was not required for the study on human participants in accordance with the local legislation and institutional requirements. The patients/participants provided their written informed consent to participate in this study.

## Author contributions

HL completed the experimental design, data collection, and calculation, as well as the writing of the paper. Both authors contributed to the article and approved the submitted version.

## Funding

This research was supported by the Research Fund of Capital University of Economics and Business (Codes: XRZ2021031 and QNTD202102).

## Conflict of interest

The authors declare that the research was conducted in the absence of any commercial or financial relationships that could be construed as a potential conflict of interest.

## Publisher's note

All claims expressed in this article are solely those of the authors and do not necessarily represent those of their affiliated organizations, or those of the publisher, the editors and the reviewers. Any product that may be evaluated in this article, or claim that may be made by its manufacturer, is not guaranteed or endorsed by the publisher.

## Footnotes

^1^This school's mission is to help diagnosed Internet addicts to quit the addiction.

^2^We did not add this data to the group of normal Internet users because we wanted to not only rely on one criterion, namely the Internet addiction test, but also to categorize participants. To be more cautious and accurately identify the two groups of participants, we wanted to use double criteria, both the diagnosis of the Internet addiction withdrawal school and the Internet addiction test.
